# Effect of in feed administration of different butyrate formulations on *Salmonella* Enteritidis colonization and cecal microbiota in broilers

**DOI:** 10.1186/s13567-020-00780-2

**Published:** 2020-04-19

**Authors:** Lonneke Onrust, Steve Baeyen, Freddy Haesebrouck, Richard Ducatelle, Filip Van Immerseel

**Affiliations:** 1 Department of Pathology, Bacteriology and Avian Diseases, Faculty of Veterinary Medicine, Ghent University, Salisburylaan 133, 9820 Merelbeke, Belgium; 2 Plant Sciences Unit, Flanders Research Institute for Agriculture, Fisheries and Food (ILVO), Merelbeke, Belgium

## Abstract

Butyrate has been used extensively as a feed additive to improve gut health and to decrease *Salmonella* colonization in poultry. *Salmonella* mainly colonizes the ceca so butyrate concentrations should be increased in this gut segment. Discrepancies on the effects of butyrate on *Salmonella* colonization, described in the scientific literature, could thus be due to butyrate release location effects. In this study, newly developed butyrate formulations were evaluated for their effect on cecal butyrate concentrations and on colonization by *Salmonella* Enteritidis. In a first trial, broilers were randomly allocated to 7 dietary treatment groups with formulations based on different approaches to modify the butyrate release profile: release from wax matrices based on diffusion/erosion; micropellets supposedly release butyrate around pH 7 in the colon; tributyrin is based on the hydrolysis of esters in the small intestine. Fat-protected butyrate was included as a reference, because of its known effect on reduction of *Salmonella* colonization. Four days after infection, the number of cfu *Salmonella* per g cecal content and spleen were determined. Butyrate formulations in a wax matrix significantly reduced the *Salmonella* colonization in cecal content. In a second trial, wax and fat-protected butyrate treatments were replicated and results from the first trial were confirmed. Compared to the control group a higher proportion of butyrate concentration was observed in ceca for those groups with reduced *Salmonella* colonization. This was associated with a beneficial shift in the cecal microbiota. In conclusion, formulations that increase cecal butyrate concentrations are superior in protecting against *Salmonella* Enteritidis colonization.

## Introduction

Despite years of strict monitoring and control in production animals worldwide, *Salmonella* is still a major food-derived zoonotic pathogen for humans. Poultry meat and eggs, as well as processed products thereof, still are the main sources of *Salmonella* infections in man [1, 2]. The most important *Salmonella* serotype associated with consumption of poultry products is *Salmonella* Enteritidis, which has been reported in more than half of the outbreaks in Europe in 2016 [1, 3]. This serotype causes egg-derived human infections, and is the consequence of systemic spread to the avian reproductive tract, resulting in internal egg contamination [4]. Poultry meat can be contaminated by a variety of serotypes, all colonizing the intestinal tract of broilers, such as serogroup C strains (e.g. Infantis), but also Typhimurium and Enteritidis. While in layers and breeders vaccination has been successful in reducing *Salmonella* Enteritidis, one is still in need for an efficient strategy to reduce gut colonization levels in broilers.

Effects of short chain fatty acids (SCFA), especially butyrate, on gastrointestinal function of animals have been widely studied over the past years [5–8]. Guilloteau et al. give an overview of the favorable effects of butyrate on the gastrointestinal tract (GIT) of broilers, including stimulation of growth performance, anti-inflammatory effects, maintenance of intestinal epithelial barrier integrity, and reduction of *Salmonella* colonization [9]. Butyrate has been extensively used as a feed additive to decrease *Salmonella* colonization in poultry in experimental models and in the field. The results, however, are not always consistent. In addition to factors such as inclusion level, diet composition, age, and health status, release locations of butyrate may partly explain the inconsistent effects of butyrate on *Salmonella* colonization [6]. Decreased intestinal *Salmonella* colonization and shedding was shown using in-feed coated butyric acid supplements, but not using uncoated butyric acid supplements [7, 10, 11], pointing to formulation effects. Unprotected butyrate is readily absorbed in the upper GIT and will not reach the ceca [12]. The ceca are the preferred colonization site of *Salmonella* and thus butyrate formulations should preferably increase butyrate concentrations in this segment [5, 7, 9]. It is striking that no studies have been published yet to determine cecal butyrate concentrations after application of in-feed butyrate supplements. Previous in vitro research has shown distinctive release profiles of newly developed and commercially available butyrate formulations, produced using different encapsulation techniques [13]. A commonly used technique is embedding butyrate in vegetable fat matrices resulting in sustained release of butyrate in the broiler GIT [14]. One of the newly developed formulations for sustained release of drugs contains a food-grade petroleum-derived wax as embedding material [14]. This technique is used in the pharmaceutical industry and shows a sustained release of butyrate in vitro [13, 15]. Direct comparisons between these formulations with respect to their effects on *Salmonella* colonization have not yet been performed.

Therefore, the purpose of the present study was to evaluate the effect of different butyrate formulations on *Salmonella* Enteritidis colonization and shedding, on the SCFA concentration in ceca, and on the cecal microbiota composition in broilers.

## Materials and methods

### Butyrate derivatives

**Figure 1:**
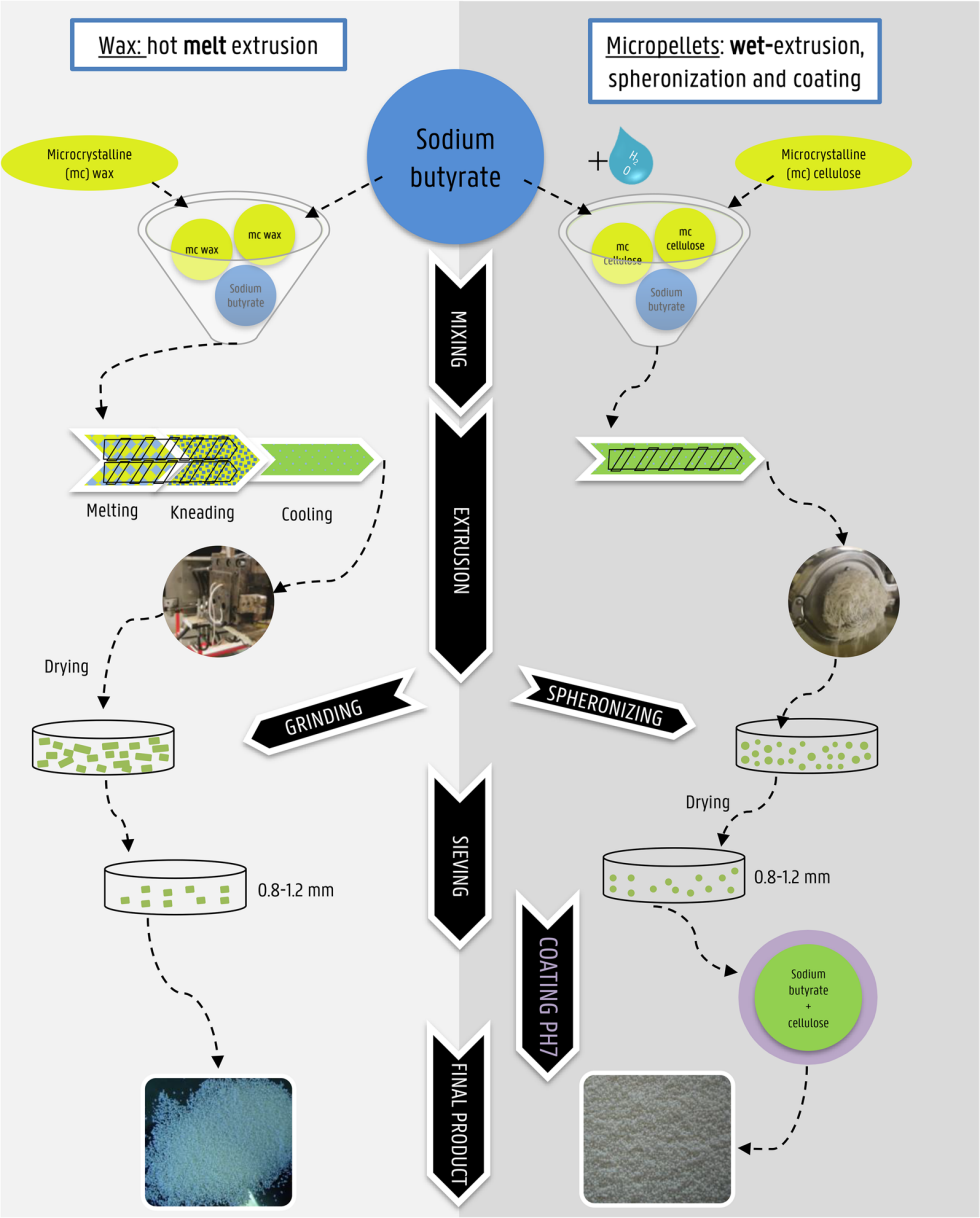
**Workflow production process novel butyrate formulations [Right] Production process of wax by using hot melt extrusion, resulting in a wax matrix carrying the active pharmaceutical ingredient sodium butyrate.** To obtain wax+ with the addition of starch, soluble potato starch in dry powder form was added during the mixing step. [Left] Production process of micropellets using wet-extrusion, spheronization and applying a pH-dependent coating. Final product is sodium butyrate with cellulose protected by an additional layer (purple sphere).

Novel butyrate derivatives were developed using different pharmaceutical technologies to produce formulations with different release patterns of butyric acid in the GIT of broilers. The first formulation consisted of a microcrystalline wax (Lunacera M wax beads, Füller GmbH, Lüneburg, Germany) and sodium butyrate (Adimix C, Nutri-Ad International NV, Dendermonde, Belgium), henceforth called Wax (resp. 70%/30% w/w). The second formulation included, in addition to the aforementioned components, S2004 soluble potato starch in dry powder form (Sigma-Aldrich, Saint-Louis, USA), hereafter called Wax + (resp. 60%/30%/10% w/w). As described by Moquet [13], melt-extrusion and grinding were used to produce matrices of microcrystalline wax containing sodium butyrate. Prior to mixing, wax beads were ground at 15 000 rpm with a ZM-1000 grinder (Retsch, Haan, Germany) equipped with a 12-tooth rotor, and without screen. Each formulation was mixed with a pedal mixer model 305 (Dinissen, Sevenum, The Netherlands) at 50% of the maximum speed for 5 min and subsequently extruded with a Baker–Perkins twin screw extruder. Extrusion settings were adjusted for each formulation in order to obtain homogenous, smooth-surfaced, non-melted extrudates [12]. Extrudates were cooled down to room temperature overnight and subsequently ground at 10 000 rpm with a ZM-1000 grinder (Retsch) equipped with a 12-tooth rotor without screen. Ground extrudates were sieved to obtain the 0.8-1.2 mm fraction (Figure 1).

A third novel butyrate formulation was developed by wet-extrusion, spheronization and fluidized-bed reactor coating, resulting in microencapsulated pellets containing butyrate with a pH-sensitive polymer, from now on called micropellets. Moquet [13] described the workflow of the production, which consisted of mixing microcrystalline cellulose (Avicel PH-101; 850 g/kg; FMC BioPolymer, Philadelphia, USA) [16] and sodium butyrate (150 g/kg; Admix C, Nutri-Ad International NV) (resp. 70%/30% w/w), and adding demineralized water in a drop-wise manner while mixing. The resulting wet mixture was extruded with a dome granulator model DG-L1 (Fuji-Paudal, Osaka, Japan) and spheronized for 5 min (spheronizer model 15, Sturminster Newton, UK). The obtained pellets were dried overnight at room temperature and sieved afterwards to select the 0.8–1.2 mm fraction. A fluidized-bed reactor model GPCG1 (Glatt GmbH, Binzen, Germany) including a Wurster module was used to coat the pellets with Eudragit^®^ FS 30 D (Evonik Industries, Essen, Germany), a copolymer based on methyl acrylate, methyl methylacrylate and methacrylic acid that dissolves at pH higher than 7 (Figure 1).

In this study, these novel butyrate derivatives were tested in parallel with a commercially available fat-protected butyrate (Adimix Precision, Nutri-ad International NV), non-protected sodium butyrate (Adimix C, Nutri-ad International NV), and tributyrin (T8626, Sigma-Aldrich). The formulations were based on different approaches to modify butyrate release profile by either sustained release or targeted release. Sustained release refers to the prolonged release of an active compound over time based on enzymatic or mechanic erosion and diffusion mechanisms [13]. Release from wax matrices is based on diffusion/erosion and is nearly pH independent. As the active compound butyrate has been embedded in the matrix (wax), no distinction can be made between an inner and outer shell. Addition of starch to the wax was used as a disintegrant, making the matrix less sustainable compared to wax and influencing the release rate of butyrate [15]. Targeted release refers to localized delivery of the active compound based on organ-specific characteristics such as pH value or enzymatic activity [13]. For the micropellets a distinction can be made between an inner and outer shell, with the outer shell a pH sensitive polymer resistant against pH values lower than 7, and the inner shell containing the active compound butyrate. As the pH increases throughout the GIT in broilers, the micropellets supposedly release butyrate around pH 7 in colon [17]. Endogenous lipases are able to cleave ester bonds of tributyrin at the first and third position of the glycerol backbone [18]. As chickens have a low pre-duodenal lipolytic activity, it is assumed that butyric acid will mostly be hydrolyzed in duodenum/jejunum by pancreatic lipases [19]. The fat-protected butyrate was included as a reference, because of its known effect on reduction of *Salmonella* colonization [7].

### Bacterial strain

*Salmonella enterica* serotype Enteritidis phage type 4 strain 147, a well characterized streptomycin resistant strain, was used in the experiments. This strain was originally isolated from egg white and has been shown to have a high capacity of colonizing the gut and internal organs in chickens [20]. The strain was grown for 6 h in Luria-Bertoni medium (LB, Sigma, St. Louis, MO, USA), after which the number of CFU (colony-forming unit) per gram was determined by plating 10-fold dilutions of the bacterial suspension on xylose lysine deoxycholate agar (XLD, Oxoid, Basingstoke, UK). The bacterial suspension was stored at 4 °C during plate counting and was diluted in Phosphate-buffered saline (PBS) to obtain the desired infection dose.

### Experimental design and diet

In a first experiment, 140-day-old male chicks were divided in 7 pens of 20 animals, assigned to 7 different dietary treatment groups. Included dietary treatments groups were given feed containing commercially available fat-protected butyrate, non-protected sodium butyrate, and the novel butyrate derivatives, i.e. wax, wax+, micropellets or tributyrin. A control group receiving non-supplemented feed was also included.

In the second experiment, only the dietary treatments which significantly reduced *Salmonella* colonization in cecum during the first trial were tested to confirm the effect on colonization. 120-day-old male chickens were divided in 6 pens of 20 animals, assigned to 3 different dietary treatment groups: non-supplemented feed, wax and commercially available fat-protected butyrate.

All feed additives were mixed in commercially available broiler feed (Versele-Laga, Deinze, Belgium) at a concentration of 3 g/kg of sodium butyrate. The experiments were approved by the ethical committee of the Faculty of Veterinary Medicine, Ghent University (EC 2014/135 and EC2015/46).

### Animals and experimental procedures

Day-old Ross-308 chicks were obtained from a local hatchery, and randomly divided in pens of 1.44 m^2^ with solid walls and a solid floor covered with fresh wood shavings. Optimal temperature for broilers was maintained during the trial, and a light schedule of 18 h light/6 h darkness was applied. The birds had ad libitum access to water and feed. At 17 days post-hatch all chicks were orally inoculated with 10^5^ CFU of *Salmonella* Enteritidis per bird. Cloacal swabs of all animals were taken the day before infection and at day 1 and 3 post-infection (dpi). At 4 dpi all birds were euthanized, and samples from cecum and spleen were taken for bacteriological analysis. Additionally, intestinal content of cecum was collected for DNA extraction and 16S rRNA sequencing. Part of the cecal content was diluted 5 times in water, and afterwards homogenized and centrifuged (2500 × *g*, 10 min) to obtain cecal water, for SCFA quantification.

### Sample processing and analysis

Bacteriological analysis was performed as described by De Cort et al. [21]. Cloacal swabs were plated directly on XLD plates supplemented with 100 μg/mL streptomycin, and enriched in buffered peptone water (Oxoid, Basingstoke, UK) overnight at 37 °C. 1 mL of this suspension was enriched by brilliant green tetrathionate broth (Merck, Darmstadt, Germany) after which plating was performed on XLD with streptomycin. Cecal samples and spleens were mechanically homogenized in buffered peptone water. Ten-fold dilutions were made in Hank’s Balanced Salt Solution (HBSS) and 6 droplets of 20 µL of each dilution were plated on XLD plates supplemented with 100 μg/mL streptomycin. After incubation overnight at 37 °C, the number of colonies was determined and numbers of CFU/g organ calculated. Samples that were negative after direct plating were enriched in buffered peptone water and brilliant green tetrathionate broth overnight at 37 °C, followed by plating on XLD. When positive after enrichment these samples were presumed to have 83 CFU/g (detection limit of direct plating). Samples that were negative after enrichment were presumed to have 0 CFU/g.

### SCFA quantification

Quantification of SCFA in cecal water was done by the method previously described by De Weirdt et al. [22]. In short, butyrate, propionate and acetate were extracted from the samples using diethylether. Extracts with methyl hexanoic acid 99% (Sigma-Aldrich) added as internal standard were analyzed on a gas chromatograph coupled with a flame-ionization detector and a split injector.

### DNA extraction

For the extraction of DNA from the pellets obtained from the cecal contents the CTAB (cetyl trimethylammonium bromide) method described by Griffiths et al. [23] and Kowalchuk et al. [24] was used. 100 mg of cecal content or 100 mg of the obtained pellets was homogenized with 0.5 mL CTAB buffer (hexadecyltrimethylammonium bromide > 98% (Sigma Aldrich) 5% (w/v), 0.35 M NaCl, 120 nM K2HPO4) and 0.5 mL phenol–chloroform-isoamyl alcohol (25:24:1) (Sigma Aldrich) in destruction tubes. After homogenization, the samples were shaken 6 times for 30 s using a beadbeater (MagnaLyser, Roche, Basel, Switzerland) at 6000 × *g* with 30 s in between shakings. The samples were centrifuged for 10 min at 8000 × *g* and 300 µL of the supernatant was transferred to a new tube. Re-extraction was done with an additional 250 µL CTAB buffer. The samples were homogenized and centrifuged again for 10 min at 8000 × *g* and 300 µL of supernatant was added to the first 300 µL. Phenol was removed by adding an equal volume of chloroform-isoamyl alcohol (24:1) (Sigma-Aldrich). The aqueous phase was transferred to a new Eppendorf tube. Nucleic acids were precipitated with 2 volumes of PEG-6000 solution (polyethyleenglycol 30% (w/v), 1.6 M NaCl). After 2 h at room temperature, a last centrifugation step was done for 20 min at 13 000 *g*. The obtained pellet was rinsed with 1 mL of ice-cold, 70% (v/v) ethanol. After drying the pellet was resuspended in 100 µL RNAse free water (VWR, Leuven, Belgium).

### 16S rRNA amplicon sequencing and processing

The 16S rRNA sequencing was performed using MiSeq v2 technology (2 × 250 bp) from Illumina at the GenoToul Genomics and Transcriptomics facility (Auzeville, France). This method has been described in detail by Vermeulen et al. [25].

Briefly, the hypervariable 16S rDNA V3–V4 region was targeted with PCR1F_460 (5′CTTTCCCTACACGACGCTCTTCCGATCTACGGRAGGCAGCAG3′) and PCR2R_460 (5′GGAGTTCAGACGTGTGCTCTTCCGATCTTACCAGGGTATCTAATCCT3′) primers. After amplification and purification, single multiplexing was performed using a 6-bp index during a second PCR with 12 cycles. Those PCR products were again purified, and the quality and the fragment length were checked before being loaded onto an Illumina MiSeq cartridge according to the manufacturer’s instructions (Illumina Inc., San Diego, CA, USA). Next, sequences were demultiplexed, trimmed, merged, filtered, and the resulting reads were clustered into Operational Taxonomic Units (OTUs) with an identity level of 97%. Chimera removal was done before the sequences of individual samples were mapped back to the representative OTUs and converted to an OTU table. OTU tables of the 16S rRNA amplicon sequencing were analyzed using the QIIME software package (v1.9.0) [26]. Bacterial OTU sequences representative for taxonomy were aligned to the Silva v119 database 97% rep set. Rarefaction analysis was done using the “alpha_rarefaction.py” script and indicated that a sequencing depth of 10 000 reads was sufficient to analyze the bacterial community in the cecal samples from broilers.

### Statistical analysis

Statistical analysis was carried out with InVivoStat (Cambridge, UK), a statistical software package which uses R as its statistics engine [27]. Model assumptions were checked by visual inspection of the residuals. Differences of the mean between dietary treatment groups were analyzed with each pen as experimental unit. The differences were considered statistically significant at *P* ≤ 0.05.

Data of the bacteriological analysis and SCFA measurements of the first trial did not meet model assumption of one-way ANOVA. Non-parametric Kruskal–Wallis test was used to analyze the data. All pairwise differences between the treatments were assessed using Behrens Fisher tests [28]. The data of the bacteriological analysis of the second trial and the SCFA measurements were assessed by one-way ANOVA using the following model: ${\text{Y}}_{ij} = \mu + \tau_{i} + \varepsilon_{ij}$, where Y_ij_ represents the *j*th replicate (*j* is 1–6) for the *i*th treatment (*i *= control, wax or fat-protected butyrate). $\mu$ is the overall mean response, τ_i_ is the *i*th treatment effect, and $\varepsilon_{ij}$ is the random error associated with the *j*th replicate fed the *i*th treatment.

For data analysis of the 16S sequencing, the OTU tables were normalized by removing those OTUs with an abundance lower than 0.01% in all samples. Multivariate analysis was done using the specific R package Vegan (version 2.0–10) [29]. Dissimilarity matrices (based on the Bray–Curtis dissimilarity index) were calculated from the OTU tables. Beta-diversity of the bacterial communities was studied by doing a Permutational multivariate analysis of variance (PERMANOVA) and a principal coordinate analysis (PCoA) on these dissimilarity indices.

To determine statistical differences in relative abundances of the bacterial families, non-parametric Kruskal–Wallis test was used to analyze the data (InVivoStat, Cambridge, UK).

## Results

### First trial: effects on *Salmonella* colonization

All cloacal swabs taken before infection were negative for *Salmonella*. One and 3 days post-infection 75% of the broilers in the control group had positive cloacal swabs. After 1 dpi 4 of the 6 dietary treatments with a butyrate derivative resulted in significantly lower numbers of animals shedding *Salmonella*, compared to the control group. Only the treatment tributyrin resulted in a significantly lower number of positive cloacal swabs at 3 dpi compared to the control group (Table 1).

**Table 1:** **Number of cloacal swabs positive for*Salmonella*Enteritidis strain 147 in the first trial**

	Dietary treatment						
	Control	Fat-protected butyrate	Uncoated butyrate	Wax	Wax+	Micropellets	Tributyrin
1 dpi	15/20	6/19	9/20	2/19*	3/20*	4/20*	2/20*
3 dpi	15/20	6/19	11/20	6/19	7/20	9/20	2/20*

Number of positive swabs on total per dietary treatment are given at 1 and 3 days post-infection (dpi) with 10^5^ CFU *Salmonella*. Broilers were fed a diet either or not supplemented with a butyrate containing feed additive in a concentration of 3 g/kg of sodium butyrate.

* Significant difference in positive samples between control and dietary treatment with butyrate derivative (*p*-value < 0.05).

**Figure 2:**
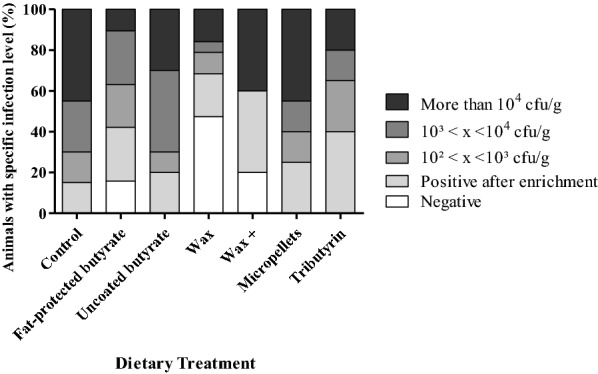
**Colonization of*Salmonella*in cecum of broilers in the first trial.** Colonization of *Salmonella* in cecum of 21-day-old broilers fed a diet either or not supplemented with a butyrate containing feed additive in a concentration of 3 g/kg of sodium butyrate. Each dietary treatment consisted of 1 pen of 20 broilers, except for fat-protected butyrate and wax it was 1 pen of 19 each due to mortality before inoculation. The bar charts are showing the percentages of animals having a specific infection level of *Salmonella* as stated in the legend (specified log number of CFU per gram cecum content).

**Figure 3:**
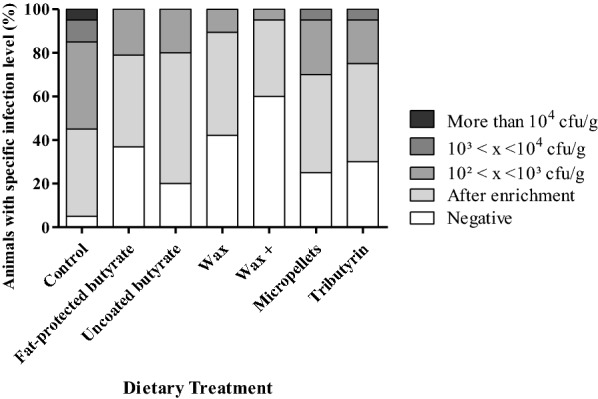
**Colonization of*Salmonella*in spleen of broilers in the first trial.** Colonization of *Salmonella* in spleen of 21-day-old broilers fed a diet either or not supplemented with a butyrate containing feed additive in a concentration of 3 g/kg of sodium butyrate. Each dietary treatment consisted of 1 pen of 20 broilers, except for fat-protected butyrate and wax it was 1 pen of 19 each due to mortality before inoculation. The bar charts are showing the percentages of animals having a specific infection level of *Salmonella* as stated in the legend (specified log number of CFU per gram spleen).

Bacteriological analysis of cecum content after 4 dpi showed that the dietary treatment group fed with the butyrate derivative wax resulted in a significantly reduced cecal colonization by *Salmonella* Enteritidis compared to the control group (*p* = 0.0006), to the groups fed a diet with unprotected sodium butyrate (*p* = 0.0018) and to the group fed micro encapsulated pellets (*p* = 0.0007) (Table 2). Figure 2 shows that the group fed a “wax” diet had a higher number of *Salmonella* negative ceca compared to the other treatment groups. No differences were detected for *Salmonella* colonization of the spleen when comparing the mean log CFU/g (Table 2) but, in general, a higher number of *Salmonella* negative spleens were found in the groups supplemented with any butyrate containing feed additive (Figure 3).

**Table 2:** **Colonization of cecum and spleen by*Salmonella*Enteritidis strain 147 in the first trial**

	Dietary treatment						
	Control	Fat-protected butyrate	Uncoated butyrate	Wax	Wax+	Micropellets	Tributyrin
Mean cecum (SD)	3.63^a^ (1.27)	2.56^ab^ (1.63)	3.45^a^ (1.15)	1.56^b^ (1.75)	2.87^ab^ (2.25)	3.87^a^ (1.76)	2.81^ab^ (1.06)
Mean spleen (SD)	2.30 (0.79)	1.34 (1.08)	1.65 (0.87)	1.18 (1.05)	0.90 (1.03)	1.68 (1.10)	1.56 (1.13)

Mean log CFU/g cecum and spleen values and standard deviation (SD) are shown at 4 dpi with 10^5^ CFU *Salmonella*. Broilers were fed a diet either or not supplemented with a butyrate containing feed additive in a concentration of 3 g/kg of sodium butyrate.

Significant differences for cecum colonization among groups are indicated with different letters (a, b). No differences for spleen colonization was detected.

### First trial: SCFA concentrations in ceca

A part of the collected cecal content at 4 dpi was used to quantify the amounts of acetate, propionate and butyrate present in ceca of the sacrificed birds. The sum of acetate, propionate and butyrate concentrations in millimolar (mM) per litre (L) are referred to as total SCFA, and is used to calculate the relative amount of the different SCFAs present in cecum. Table 3 gives an overview of those concentrations in mM and relative amounts of SCFA per treatment group, including differences between the treatment groups. In the text below only the differences with the control group are mentioned.

**Table 3:** **Concentrations of SCFAs measured in cecum of broilers in the first trial**

	Dietary treatment						
	Control	Fat-protected butyrate	Uncoated butyrate	Wax	Wax+	Micro pellets	Tributyrin
Butyrate (mM) (SD)	9.38^a^ (3.58)	13.00^a^ (3.01)	9.22^a^ (4.69)	12.93^a^ (7.45)	12.37^a^ (3.09)	11.45^a^ (3.90)	9.23^a^ (4.67)
% butyrate/total SCFA (SD)	14.95^ace^ (3.77)	17.93^be^ (2.83)	14.81^a^ (5.35)	25.15^d^ (5.88)	18.26^bc^ (2.22)	21.29^bd^ (5.66)	16.41^ab^ (6.00)
Acetate (mM) (SD)	51.52^ab^ (18.48)	56.00^a^ (9.02)	50.11^ab^ (12.67)	38.60^ab^ (23.44)	53.94^ab^ (13.09)	41.92^b^ (14.10)	42.38^b^ (10.01)
% acetate/total SCFA (SD)	81.43^b^ (13.29)	79.11^bc^ (3.40)	81.18^b^ (5.27)	72.18^a^ (5.45)	79.26^bc^ (2.66)	76.43^ac^ (5.22)	78.55^bc^ (5.46)
Propionate (mM) (SD)	2.28^bd^ (0.99)	2.09^ab^ (1.11)	2.54^bd^ (0.97)	1.31^ac^ (0.95)	1.65^acd^ (0.76)	1.23^c^ (0.68)	2.54^b^ (0.97)
% propionate/total SCFA (SD)	4.17^ac^ (1.93)	2.97^bc^ (1.58)	4.01^ac^ (2.23)	2.67^acd^ (1.36)	2.47 ^cd^ (1.13)	2.28^bd^ (0.99)	5.04^a^ (2.54)
Total SCFA (SD)	63.18^ab^ (21.01)	71.09^b^ (11.15)	63.34^ab^ (15.12)	52.84^ab^ (29.62)	67.96^ab^ (15.45)	54.6^a^ (16.35)	54.15^a^ (12.67)

The broilers were fed a diet either or not supplemented with a butyrate containing feed additive in a concentration of 3 g/kg of sodium butyrate radical. Each dietary treatment consisted of 1 pen of 20 broilers. Measurements of SCFA concentrations were done at the age of 21 days after 4 days of *Salmonella* infection.

Significant differences for SCFA concentrations or percentages among dietary treatments are indicated with different letters per row (a, b, c, d, e).

The absolute butyrate concentrations in mM/L didn’t show any differences between the treatment groups, but when comparing the relative amounts of butyrate (% butyrate/total SCFA) present in ceca, the group fed a wax diet and micropellets had a significantly higher percentage cecal butyrate as compared to the control group (resp. *p* < 0.0001 and *p* = 0.0043).

Comparing the relative amounts of acetate concentrations (%) showed that the wax group and the micropellet group had a significantly lower percentage of acetate compared to the control group (resp. *p* < 0.0001 and *p* = 0.0365).

The propionate concentrations (mM/L) were lower in the wax and micropellet group compared to the control group (resp. *p* = 0.0045, *p* = 0.0004). The relative amount of propionate present was only lower in micropellet group compared to the control group (*p* = 0.0034).

### Second trial: effects on *Salmonella* colonization

In the second trial only the butyrate derivatives that reduced *Salmonella* colonization most significantly in the first trial were tested again (fat-protected butyrate and wax). All cloacal swabs taken before infection were negative for *Salmonella*, and no significant differences were detected in number of positive cloacal swabs after 1 (*p* = 0.0626) and 3 dpi (*p* = 0.0513) (Table 4).

**Table 4:** **Number of cloacal swabs positive for*Salmonella*Enteritidis strain 147 in the second trial**

	Dietary treatment		
	Control	Fat-protected butyrate	Wax
1 dpi	23/39	18/40	13/40
3 dpi	30/39	22/40	21/40

Number of positive swabs on total per dietary treatment are given at 1 and 3 days post-infection (dpi) with 10^5^ CFU *Salmonella*. Broilers were fed a diet either or not supplemented with a butyrate containing feed additive in a concentration of 3 g/kg of sodium butyrate.

**Figure 4:**
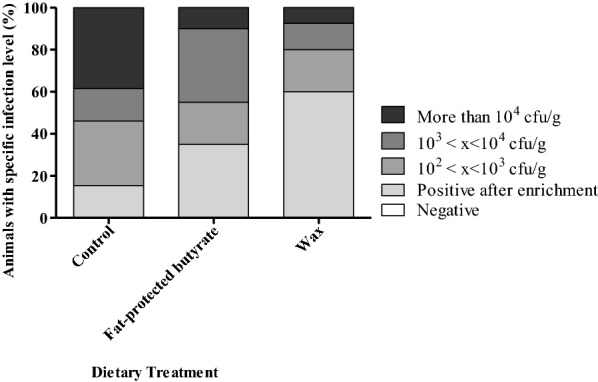
**Colonization of*Salmonella*in ceca of broilers in the second trial.** Colonization of *Salmonella* in ceca of 21-day-old broilers fed a diet either or not supplemented with a butyrate containing feed additive in a concentration of 3 g/kg of sodium butyrate. Each dietary treatment consisted of 2 pens of 20 broilers. The bar charts are showing the percentages of animals having a specific infection level of *Salmonella* as stated in the legend (specified log number of CFU per gram cecum content).

**Figure 5:**
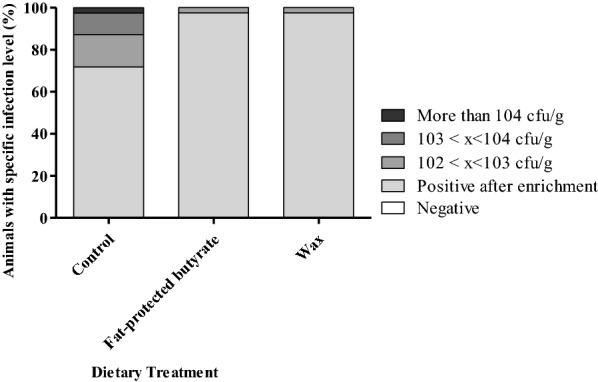
**Colonization of*Salmonella*in spleen of broilers in the second trial.** Colonization of *Salmonella* in spleen of 21-day-old broilers fed a diet either or not supplemented with a butyrate containing feed additive in a concentration of 3 g/kg of sodium butyrate. Each dietary treatment consisted of 2 pens of 20 broilers. The bar charts are showing the percentages of animals having a specific infection level of *Salmonella* as stated in the legend (specified log number of CFU per gram spleen content).

Bacteriological analysis of cecal content after 4 dpi showed that the dietary treatment group fed with wax had a significantly reduced cecal colonization by *Salmonella* Enteritidis compared to the control group (*p* < 0.0001) (Table 5). Figure 4 shows that the broilers fed a “wax” diet had a lower number of *Salmonella* positive ceca after direct plating compared to the other treatment groups. Bacterial counts in spleen revealed no differences in *Salmonella* colonization comparing the mean log CFU/g (Table 5), but in general a lower number of caeca were found to have *Salmonella* positive samples after direct plating in the groups supplemented with a butyrate containing feed additive (Figure 5).

**Table 5:** **Colonization of cecum and spleen by*Salmonella*Enteritidis strain 147 in the second trial**

	Dietary treatment		
	Control	Fat-protected butyrate	Wax
Mean cecum (SD)	3.64^a^ (1.47)	2.89^ab^ (1.04)	2.40^b^ (0.76)
Mean spleen (SD)	2.25 (0.64)	1.93 (0.08)	1.94 (0.12)

Mean log CFU/g cecum and spleen values and standard deviation (SD) are shown at 4 dpi with 10^5^ CFU *Salmonella*. Broilers were fed a diet either or not supplemented with a butyrate containing feed additive in a concentration of 3 g/kg of sodium butyrate.

Significant differences for cecum colonization among groups are indicated with different letters (a, b). No differences for spleen colonization was detected.

### Second trial: SCFA concentrations in cecum

Fat-protected butyrate yielded the highest total SCFA concentration of all tested groups, which was significantly higher compared to the wax treatment (*p* = 0.0008). Table 6 gives an overview of those concentrations in mM and relative amounts of SCFA per treatment group, including differences between the treatment groups.

**Table 6:** **Concentrations of SCFAs measured in cecum of broilers in the second trial**

	Dietary treatment		
	Control	Fat-protected butyrate	Wax
Butyrate (mM) (SD)	8.48^a^ (2.69)	12.45^b^ (3.94)	13.45^b^ (3.32)
% butyrate/total SCFA (SD)	15.02^a^ (2.75)	18.39^b^ (2.89)	27.16^c^ (4.37)
Acetate (mM) (SD)	47.55^a^ (14.12)	52.2^a^ (9.74)	34.4^b^ (7.65)
% acetate/total SCFA (SD)	82.70^a^ (3.46)	78.71^b^ (2.80)	69.09^c^ (4.45)
Propionate (mM) (SD)	1.25^a^ (0.51)	1.87^b^ (0.69)	1.83^b^ (0.56)
% propionate/total SCFA (SD)	2.29^a^ (1.00)	2.89^ab^ (1.20)	3.75^b^ (1.28)
Total SCFA (SD)	57.29^ab^ (16.4)	67.27^a^ (13.23)	49.67^b^ (9.80)

The broilers were fed a diet either or not supplemented with a butyrate containing feed additive in a concentration of 3 g/kg of sodium butyrate radical. Each dietary treatment consisted of 2 pens of 20 broilers. Measurements of SCFA concentrations were done at the age of 21 days after 4 days of *Salmonella* infection.

Significant differences for SCFA concentrations or percentages among dietary treatments are indicated with different letters per row (a, b).

Both absolute (mM) as well as relative (%) butyrate concentrations were significantly higher for both treatment groups compared to the control group [control vs fat-protected butyrate (mM *p* = 0.0017, % *p* = 0.0096), control vs wax (mM and % *p* < 0.0001)]). The relative amount of butyrate was higher for the wax group compared to the fat-protected butyrate group (*p* < 0.0001).

The wax treatment group had a higher butyrate concentration, but a significantly lower acetate and propionate concentration compared to the control group, both in absolute and relative numbers (mM acetate *p* = 0.0013, % acetate *p* < 0.0001, mM propionate *p* = 0.0116; % propionate *p* = 0.0008). Fat-protected butyrate yielded a higher concentration of acetate relative to the total SCFA and in mM compared to wax (*p* < 0.0001), and relatively higher percentage of acetate compared to the control group (*p* = 0.0037). Only in absolute numbers the propionate concentration was higher in the fat-protected butyrate group compared to the control group (*p* = 0.0058).

### 16S rDNA V3–V4 sequencing

**Figure 6:**
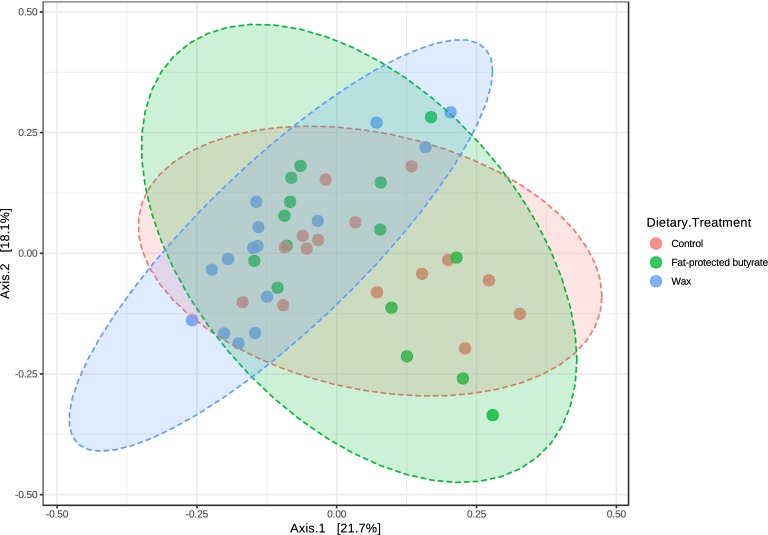
**Diversity measures (beta-diversity) for the cecal bacterial communities in the second trial.** Diversity measures (beta-diversity) for the cecal bacterial communities of chickens in the control group, and the groups receiving wax and fat-protected butyrate. Cecal content was collected from the 21-day-old chickens in the first trial, 4 days after *Salmonella* infection, while receiving a standard diet supplemented with wax (blue) or fat-protected butyrate (green) or non-supplemented (pink) for 21 consecutive days. The PCoA plot of the abundance based on Bray–Curtis dissimilarity matrix showed diet-related clustering indicated with ellipses with *p*-value < 0.001, and was created with the web-based tool MicrobiomeAnalyst [47].

**Figure 7:**
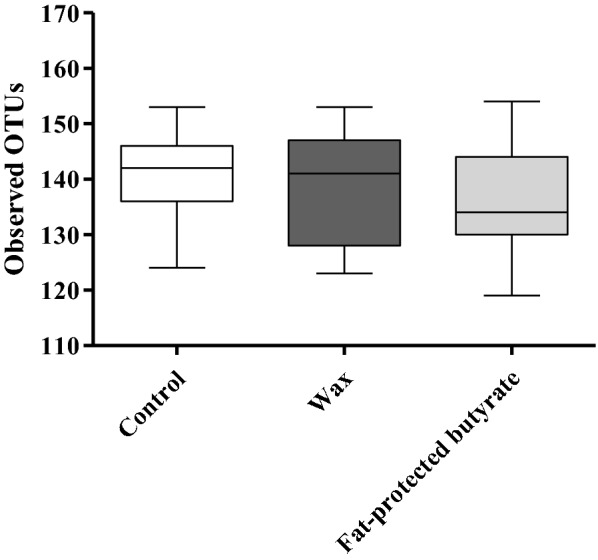
**Richness measures (alpha diversity) for the cecal bacterial communities in the second trial.** The richness has been visualized for the control group and the group receiving wax and fat-protected butyrate. Cecal content was collected from the 21-day-old chickens in the first trial 4 days after *Salmonella* infection and receiving a standard diet supplemented with wax or fat-protected butyrate or non-supplemented diet for 21 consecutive days. The horizontal lines at the bottom, the middle and the top of the box represent the first quartile, median and third quartile, respectively. The whiskers indicate the min/max value.

Cecal content was collected from the 21-day-old chickens in the first trial, 4 days after *Salmonella* infection, in groups receiving a standard diet supplemented with wax or fat-protected butyrate, or non-supplemented diet for 21 consecutive days. DNA was extracted from the cecal content, and samples were sent for Illumina sequencing. Diversity or beta diversity of the cecal bacterial communities of chickens in the control group and the group receiving wax and fat-protected butyrate are shown in a PCoA plot based on the Bray–Curtis dissimilarity index (Figure 6). The PCoA plot of the abundance based on Bray–Curtis dissimilarity matrix showed diet-related clustering (*p* < 0.001). Analyzing the alpha diversity, or richness, of the cecal content, expressed as the number of observed OTUs, revealed no significant differences between the dietary treatment groups (Figure 7).

**Figure 8:**
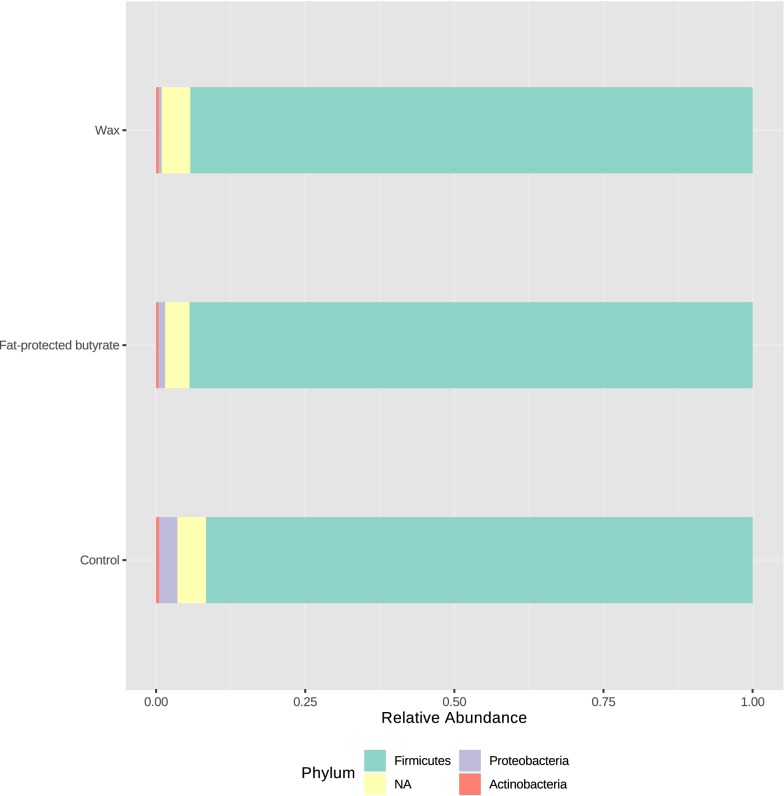
**Relative abundance of the most important bacterial phyla.** Visualization of the relative abundance of the most important bacterial phyla present in the dietary treatment groups. Cecal content was collected from the 21-day-old chickens in the first trial 4 days after *Salmonella* infection and receiving a standard diet supplemented with wax or fat-protected butyrate or non-supplemented for 21 consecutive days. DNA was extracted from the cecal content, and relative abundances are shown as determined by Illumina sequencing, and visualized with the web-based tool MicrobiomeAnalyst [47].

Members of the phylum Proteobacteria were significantly decreased in cecal contents from chickens that received the diet containing wax (0.6% *p* = 0.0004) or fat-protected butyrate (0.9% *p* = 0.0037) compared to the non-supplemented group (3.2%) (Figure 8). The same reduction was observed at family and genus level for resp. *Enterobacteriaceae* and *Escherichia/Shigella* (Table 7).

**Table 7:** **Relative abundances of bacterial phyla, families and genera in the microbial community in ceca of broilers**

Phylum	Family	Genus	Dietary treatment					
			Control		Wax		Fat-protected butyrate	
			Mean	SD	Mean	SD	Mean	SD
Firmicutes			91.63^a^	6.10	93.94^a^	2.92	94.57^a^	2.73
	*Lachnospiraceae*		52.75^a^	12.17	62.48^b^	6.13	50.68^a^	11.56
		*Blautia*	12.12^a^	4.26	16.44^b^	4.87	12.96^ab^	4.51
		*Other/uncultured*	29.80^a^	10.22	37.83^b^	7.05	29.72^ab^	11.06
	*Ruminococcaceae*		18.38^ab^	9.15	13.85^a^	4.85	24.15^b^	9.19
		*Subdoligranulum*	4.56^a^	7.44	0.31^b^	0.41	5.85^ab^	10.65
		*Anaerotruncus*	2.55^a^	1.66	2.23^a^	1.51	2.98^a^	1.65
		*Other/uncultured*	11.03^a^	4.18	11.19^a^	4.04	14.69^a^	5.16
	*Lactobacillaceae*		10.22^a^	8.19	3.82^b^	3.86	7.54^ab^	6.14
		*Lactobacillus*	10.22^a^	8.19	3.82^b^	3.86	7.54^ab^	6.14
	*Streptococcaceae*		7.91^a^	4.54	8.34^a^	4.95	7.96^a^	3.00
	*VadinBB60*		1.14^a^	1.02	3.62^b^	2.33	2.44^ab^	2.77
	*Defluviitaleaceae*		0.15^a^	0.05	0.30^b^	0.16	0.03^b^	0.14
	*Peptoniphilaceae*		0.05^a^	0.04	0.01^b^	0.02	0.00^b^	0.01
	*Lachnospiraceae *+* Ruminococcaceae*		71.13^a^	8.11	76.33^a^	6.84	74.84^a^	7.19
Proteobacteria			3.16^a^	5.28	0.56^b^	0.45	0.91^b^	1.32
	*Enterobacteriaceae*		1.80^a^	1.21	0.54^b^	0.44	0.29^b^	1.32
		*Escherichia/Shigella*	1.76^a^	1.17	0.54^b^	0.44	0.88^b^	1.33
Actinobacteria			91.64^a^	6.10	93.94^a^	2.92	94.58^a^	2.73
Other			4.69^a^	3.58	5.03^a^	2.87	4.11^a^	2.20

At the age of 21 days after 4 days of *Salmonella* infection relative abundances in cecal content were determined with 16S rRNA V3–V4 amplicon sequencing. The broilers were fed a diet either or not supplemented with a butyrate containing feed additive in a concentration of 3 g/kg of sodium butyrate. Each dietary treatment consisted of 2 pens of 20 broilers.

Significant differences for relative abundances of bacterial phyla, families and genera among dietary treatments are indicated with different letters per row (a, b).

**Figure 9:**
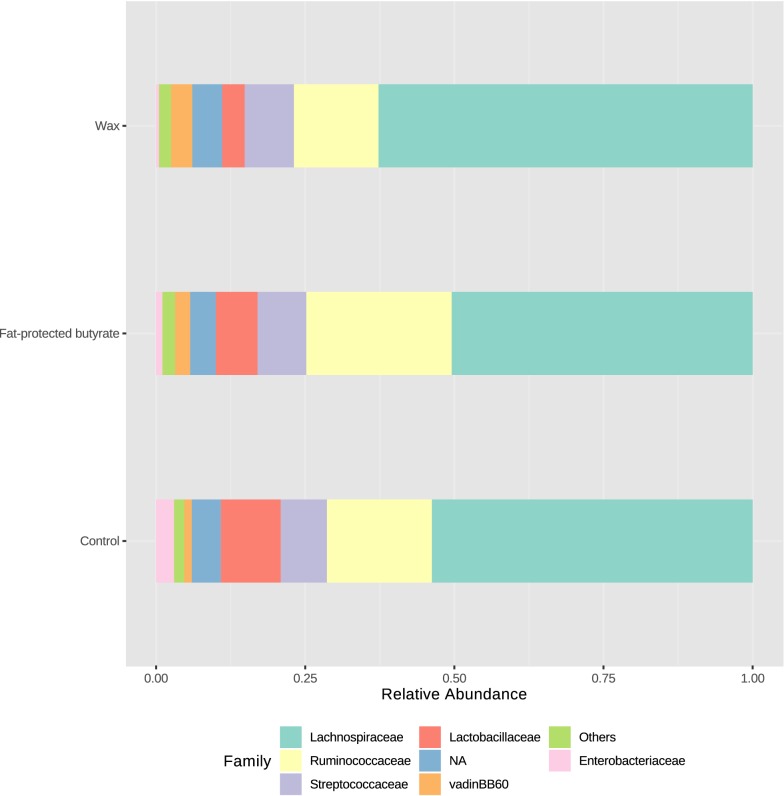
**Relative abundance of the most abundant bacterial families.** Visualization of the relative abundance of the most abundant bacterial families present in the caeca of animals in the different dietary treatment groups. Cecal content was collected from the 21-day-old chickens in the first trial 4 days after *Salmonella* infection and receiving a standard diet supplemented with wax or fat-protected butyrate or non-supplemented for 21 consecutive days. DNA was extracted from the cecal content, and relative abundances are shown as determined by Illumina sequencing, and visualized with the web-based tool MicrobiomeAnalyst [47].

Figure 9 shows the relative abundances observed at family level. The most abundant family in analyzed samples was *Lachnospiraceae*, which was significantly increased in the wax group (62.5%) compared to the control group (52.8% *p* = 0.0181). A large proportion of this family was assigned to uncultured genera. Those OTUs together with the ones assigned to genus *Blautia* were responsible for the shift to *Lachnospiraceae* in the wax group (Table 7).

Although no reduction of the abundance of the family *Ruminococcaceae* could be observed for the wax group compared to the control group (*p* = 0.0815), the genus *Subdoligranulum* within this family was significantly decreased in the wax group (0.3%) compared to the control group (4.6%, *p* = 0.0169) (Table 7).

The relative abundance of *Lactobacillaceae* was significantly decreased for the wax group (3.8%) versus the control group (10.2% *p* = 0.0028), which was solely due to the reduction of the genus *Lactobacillus*.

Members of the family VadinBB60 were increased in abundance for the group receiving a diet containing wax (3.6%) compared to the control group (1.1% *p* = 0.0021).

Other significant differences were observed at family level with lower abundances for the families *Defluviitaleaceae* and *Peptoniphilaceae* (Table 7).

## Discussion

One of the challenges in the use of butyrate as feed additive is having a reliable system to deliver the molecule to the preferred location in the intestinal tract. Mainly two techniques can be found in the literature, being esters composed of butyric acid and glycerol, and butyrate embedded in a matrix of vegetable fat developed by spray cooling or film coating. The first is believed to yield higher butyrate concentrations in the upper GI tract while the latter would carry butyrate further down the GI tract, although data on effective butyrate concentrations in the gut of poultry are scarce in the literature [30]. In the pharmaceutical field one of the strategies to bring active compounds past the gastric section in the GIT and induce a sustained release, is production of matrix pellets by melt granulation based on microcrystalline waxes and starch derivatives. Drug release can be influenced by the processing parameters as well as the amount of starch due to change of matrix solubility [15]. Another strategy to bypass the stomach for orally administered pharmaceuticals, is the production of coated pellets by extrusion and spheronization and applying an enteric coating. The type of coating determines the drug release, and plays an important role in protecting drugs that are decomposable in the stomach by low pH or enzymatic degradation. A very site-specific release of the drug can be obtained with different types of coatings based on pH in the GIT, such as coated pellets for colon delivery with a coating soluble at pH 7 or higher [31].

Previous in vitro research showed that the matrix pellets based on microcrystalline wax had a sustained release profile with an increased release of butyrate at the simulated ileum. Commercially available fat-coated butyrate products with a different production process than the wax matrices (spray chilling vs hot melt extrusion) showed variations in release properties. Only two of the commercially tested products had an extended release profile in vitro. In previous work, micropellets showed a targeted release profile. They were partially protected during gastric passage, followed by a rapid release in the enteric segments reaching pH 6.5 which was expected with the type of coating used [13]. We investigated the potential of newly developed butyrate derivatives and one commercially available fat-coated butyrate product with extended release profile to increase butyrate concentration in the ceca and to reduce colonization of *Salmonella* in the ceca. It was hypothesized that a more sustained release and an increase in cecal butyrate concentrations would decrease *Salmonella* colonization in the ceca. The butyrate effect on *Salmonella* is through suppression of invasion of *Salmonella* in epithelial cells and consequently gut colonization [32]. One of the important transcriptional activators of *Salmonella* pathogenicity island 1 (SPI1) that regulates the invasion of *Salmonella* is the *hilA* gene [33]. Mutants in this gene have been shown to be poor gut colonizers [34]. Previously downregulation of *hilA* gene after exposure to butyrate and propionate was reported, while expression increased after exposure of the bacteria to acetate [32, 35]. In our study we showed that in the ceca of chickens fed a wax matrix the relative proportion of butyrate increased, while acetate proportion was reduced compared to the control group. This shift in SCFA proportions indicates a less favorable environment for *Salmonella* to invade the epithelial cells of the cecal mucosa increasing the resilience of chickens against *Salmonella* infections. The results of increased SCFA concentrations in the hindgut are in line with the findings of Van den Borne [14], but in contrast with the research of Moquet et al. where no statistical differences could be observed in cecum and colon concentrations, but only numerical differences after feeding with the same fat-protected butyrate (although a large variation within the groups was reported) [13]. Although the birds were roughly the same age, the type of challenge may play a role in these discrepancies, namely bacterial challenge by *Salmonella* infection versus a dietary challenge induced by rapeseed meal diet.

Literature indicates that supplementing diets of broilers with butyrate influences cecal microbiota composition in a way that is beneficial for the health and growth performance when the microbiota is disturbed by for example an enteric disease or nutritional challenge [36–38]. We also observed that supplementing butyrate in a wax-based carrier can reduce the *Salmonella* count, which very likely explains the observed decrease of *Enterobacteriaceae*. We observed that wax coated butyrate increased the relative abundance of *Lachnospiraceae*, a family containing important butyrate-producing bacteria. Besides an increase of genera within this family that are not yet cultured, members of the genus *Blautia* were also increased with wax supplementation. Species belonging to *Blautia* can use carbohydrates as a substrate to produce lactate and acetate as the major end product of glucose fermentation [39, 40]. They are associated with a reduction of incidence of inflammatory bowel disease in humans [41]. Another family with important butyrate-producers is *Ruminococcaceae*. *Subdoligranulum*, one of the genera in this family that has butyric and lactic acid as major end products of fermentation, was decreased in abundance after wax supplementation in this study [42]. Analyzing the sum of both families resulted in no differences between the dietary treatment groups. Accordingly, it is not clear whether the observed increased concentrations in cecal butyrate in the supplemented groups are mainly due to the exogenous butyrate or whether endogenous butyrate is also adding on top of this.

In our study a significant decrease of cecal *Lactobacillaceae* was observed when butyrate was added to the diet in a wax or fat-coated form. *Lactobacillus* spp. are probably the most commonly used probiotics and they are linked to several benefits for intestinal health [43]. Several other studies with butyrate supplementation reported the same observation regarding *Lactobacillus* reductions, both in broilers in cecal lumen as well as in weaned piglets in ileal and colonic lumen [44, 45]. Based on a previous study by De Boever et al. showing that *Lactobacillus reuteri* plays an active role in microbial bile salt hydrolase production resulting in impaired lipid absorption and therefore dietary energy losses, it is hypothesized that a reduction of *Lactobacillaceae* can play a role in reduced energy loss, and thus increases nutrient utilization with a better feed conversion ratio as profitable result [46]. In a recent study in pigs, a better feed efficiency was correlated amongst others with an increase of *Clostridiales* genus VadinBB60 [46]. In our study a higher abundance of VadinBB60 was observed with wax dietary treatment, but feed conversion ratio was not evaluated.

In conclusion, the newly developed microcrystalline wax matrix as carrier for sodium butyrate may be a suitable feed supplement to protect broilers against *Salmonella* Enteritidis. In this study the matrix was tested against a single strain *Salmonella* Enteritidis 147 Strep, which is known to colonize the gut and internal organs of chickens to a high level. Compared to other carriers, the delayed release of butyrate induced a higher relative cecal butyrate concentration and a decrease of *Salmonella* colonization in ceca. The dietary treatment of wax containing sodium butyrate modulated the cecal microbiota of the challenged chickens.

## Data Availability

All data generated during this study are included in this published article. The datasets used and/or analyzed during the current study are available from the corresponding author on reasonable request.

## Abbreviations

CFU: colony-forming unit
PBS: Phosphate-buffered saline
SCFA: short chain fatty acids
GIT: gastro intestinal tract
mc: microcrystalline
mM: millimolair
XLD: xylose lysine deoxycholate
HBSS: Hank’s Balanced Salt Solution
DNA: Deoxyribonucleic acid
rDNA: Ribosomal deoxyribonucleic acid
CTAB: cetyl trimethylammonium bromide
PEG: polyethyleenglycol
OTU: Operational taxonomic unit
ANOVA: Analysis of variance
PERMANOVA: Permutational multivariate analysis of variance
PCoA: principal coordinate analysis
